# The complete mitochondrial genome sequence of an alpine plant *Arabis alpina*

**DOI:** 10.1080/23802359.2018.1483758

**Published:** 2018-06-29

**Authors:** Yiqing Xu, Changwei Bi

**Affiliations:** a School of Computer Science and Engineering, Southeast University, Nanjing, Jiangsu, China;; b School of Biological Science and Medical Engineering, Southeast University, Nanjing, Jiangsu, China

**Keywords:** *Arabis alpina*, mitochondrial genome, alpine plant, phylogeny

## Abstract

*Arabis alpina* (*A. alpina*) is an arctic-alpine flowering plant in the family Brassicaceae, naturally growing in the tundra of arctic regions and in mountains at southern latitudes. In this study, we first report the assembly of the complete *A. alpina* mitochondrial (mt) genome using the next-generation sequencing technologies. The *A. alpina* mt circular genome is 323,159 bp in length and contains 33 protein-coding genes, 18 tRNA genes and 3 rRNA genes. To analyze the phylogenic and evolutional relationship of *A. alpina*, a neighbour-joining phylogenetic tree was reconstructed based on the mt genome of *A. alpina* and other 27 plants. The complete *A. alpina* mt genome will be helpful in population studies or investigations of mt functions of these alpine plant species.

The alpine plant *Arabis alpina* is a thoroughly studied plant, which is well suited to analyze the flowering regulation of perennial plants (Bergonzi et al. 2013), plant phylogeography (Karl et al. 2012) and local adaptation to harsh alpine environmental conditions (Zulliger et al. 2013). Recently, there is growing interest to develop *A. alpina* as an emerging model for the study of genetics, population genetics and molecular biology (Lobréaux et al. 2014).

In most eukaryotic organisms, mitochondria exist as a double-membrane-bound organelle, which generate most of the cell’s supply of ATP, and used as a source of chemical energy (Ye et al. 2017). It has been demonstrated that plant mt genomes vary considerably in their structure, length and gene order (Bi et al. 2016). Therefore, it is difficult to de novo assemble a plant mt genome. In this study, we assembled the complete *A. alpina* mt DNA sequence into a circular genome, which may contribute to improving our understanding of the population studies or investigations of mt functions in alpine plant organisms.

The plant sample of *A. alpina* used for extraction and genomic sequencing was collected in the French Alpsat site 11 in the Vercors mountains (Geographic coordinate: 44°52′47″N, 5°31′21″E, Elevation: 2010 m). Genomic DNA was extracted from 20 mg of dried material ground into a fine powder following the manufacturer’s instructions, and then the DNA was deposited in Laboratoire d’Ecologie Alpine of Université Joseph Fourier (Grenoble, France). All genomic DNA samples were sequenced using Illumina technology and Roche/454 GSFLX Titanium, and the generated sequencing reads were a mixture of nucleus, chloroplast and mitochondrion. In order to assemble a complete *A. alpina* mt genome, we first assembled the mixed Roche/454 generated reads using Newbler 3.0 (Roche Diagnostics company, Indianapolis, IN) with default parameters. After that, we filtered the mt contigs with the depth between 20 and 50, and linked these filtered contigs based on the Newbler generated file ‘454AllContigGraph.txt’ using in-house Perl scripts. Gaps and errors in assembled contigs were filled up and corrected by Illumina generated reads with the software BWA (Li 2013) and MacVector. Finally, a total of 323,159 bp nucleotide mt genome were completely assembled, and then submitted to GenBank with the accession number NC_037070.1. The overall base composition of the *A. alpina* mt genome is A: 27.7%, G: 22.53%, C: 22.41%, T: 27.36%, and the GC content is 44.94%.

The *A. alpina* mt genome was annotated with an online grogram MITOFY (Alverson et al. 2010), and a total of 54 genes were identified, composed of 33 protein-coding genes, 18 tRNA genes and 3 rRNA genes. Among the 33 protein-coding genes, 9 genes (*ccmFc*, *cox2*, *nad1*, *nad2*, *nad4*, *nad5*, *nad7*, *rpl2* and *rps3*) were identified to contain a total of 18 introns and 32 exons. Additionally, we analyzed the codon usage of these protein-coding genes, and the results showed that most of the genes shared the same start codon ATG, except *nad1* uses ACG as start codon because of C to U RNA-editing, and two genes (*matR* and *mttB*) have no start codons probably due to the base mutation in evolution. Three types of stop codons were also identified in these protein-coding genes: TAG (*atp1*, *nad7*, *rps3* and *matR*), TGA (*atp8*, *atp9*, *cox3*, *ccmC*, *ccmFn*, *cob*, *nad4*, *rps12*, *rpl2* and *mttB*), TAA (*atp4*, *atp6*, *cox1*, *cox2*, *ccmB*, *ccmFc*, *nad1*, *nad2*, *nad3*, *nad4L*, *nad5*, *nad6*, *nad9*, *rps4*, *rps7*, *rpl5* and *rpl16*). In order to confirm the phylogenetic position of the *A. alpina* mt genome, 23 conserved protein-coding genes (*atp1*, *atp4*, *atp6*, *atp8*, *atp9*, *ccmB*, *ccmC*, *ccmFc*, *ccmFn*, *cob*, *cox1*, *cox2*, *cox3*, *nad1*, *nad2*, *nad3*, *nad4*, *nad4L*, *nad5*, *nad6*, *nad7*, *nad9* and *matR*) were extracted from 28 plant mt genomes to reconstruct the phylogenetic tree. *Cycas taitungensis* and *Ginkgo biloba* were set as out-group. As illustrated in Figure 1, the neighbour-joining phylogenetic tree exhibited that *A. alpina* mt genome was evolutionarily close to that of *Brassica napus* and then *Arabidopsis thaliana* in Brassicaceae family. The complete mt genome of *A. alpina* would contribute to the further biological study of mt genomes in Brassicaceae and provide vital functional information of *A. alpina* in ecology.

**Figure 1.**
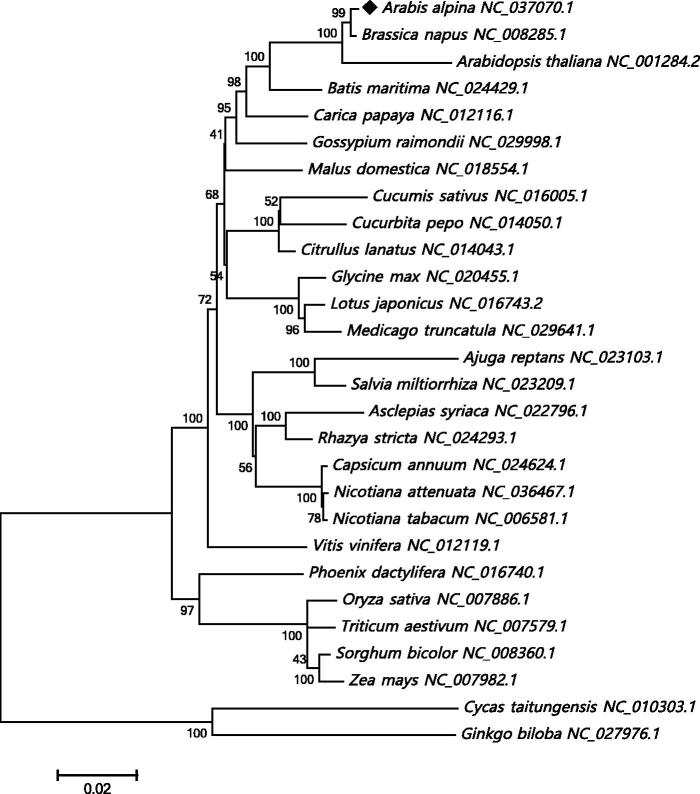
The neighbour-joining phylogenetic tree of 28 plant mt genomes based on 23 conserved mt genes. Bootstrap values are listed for each node. Accession numbers for tree construction are listed right to their scientific names.
